# Do TUNEL and Other Apoptosis Assays Detect Cell Death in Preclinical Studies?

**DOI:** 10.3390/ijms21239090

**Published:** 2020-11-29

**Authors:** Razmik Mirzayans, David Murray

**Affiliations:** Department of Oncology, Cross Cancer Institute, University of Alberta, Edmonton, AB T6G 1Z2, Canada; david.murray5@ahs.ca

**Keywords:** apoptosis, reversal, anastasis, DNA strand breakage, TUNEL, polyploid giant cancer cells, micronucleation, senescence, cancer therapy

## Abstract

The terminal deoxynucleotidyl transferase-mediated dUTP nick end labeling (TUNEL) assay detects DNA breakage by labeling the free 3ʹ-hydroxyl termini. Given that genomic DNA breaks arise during early and late stages of apoptosis, TUNEL staining continues to be widely used as a measure of apoptotic cell death. The advantages of the assay include its relative ease of performance and the broad availability of TUNEL assay kits for various applications, such as single-cell analysis of apoptosis in cell cultures and tissue samples. However, as briefly discussed herein, aside from some concerns relating to the specificity of the TUNEL assay itself, it was demonstrated some twenty years ago that the early stages of apoptosis, detected by TUNEL, can be reversed. More recently, compelling evidence from different biological systems has revealed that cells can recover from even late stage apoptosis through a process called anastasis. Specifically, such recovery has been observed in cells exhibiting caspase activation, genomic DNA breakage, phosphatidylserine externalization, and formation of apoptotic bodies. Furthermore, there is solid evidence demonstrating that apoptotic cells can promote neighboring tumor cell repopulation (e.g., through caspase-3-mediated secretion of prostaglandin E_2_) and confer resistance to anticancer therapy. Accordingly, caution should be exercised in the interpretation of results obtained by the TUNEL and other apoptosis assays (e.g., caspase activation) in terms of apoptotic cell demise.

## 1. Introduction

The Nomenclature Committee on Cell Death (NCCD) has formulated several caveats concerning the misuse of words and concepts that have slowed down progress in the area of cell death research. In their 2009 article [1], the NCCD encouraged authors, reviewers and editors of scientific periodicals to abandon expressions, like “percentage apoptosis” and “percent survival”, and to replace them with more realistic descriptors for the specific biochemical and cellular parameters that are actually being measured, such as “percent cleaved caspase-3 positive,” “percent terminal deoxynucleotidyl transferase-mediated dUTP nick end labeling (TUNEL) positive,” “percent propidium iodide positive”, and “percent clone forming” cells. Since then, numerous other groups have highlighted the potential misinformation perpetuated by the misguided use of cell-based preclinical assays for assessment of cancer cell death; these include large dye uptake assays [2,3], multiwell plate “viability” or “cytotoxicity” assays [3,4,5,6,7], and the colony formation assay [4,6,7]. Unfortunately, the NCCD guidelines and similar cautionary recommendations by others continue to be overlooked in numerous cancer therapy-related publications.

Given that assessment of the cleavage of genomic DNA induced by anticancer agents, detected by TUNEL, continues to be widely used as a measure of cancer cell death by apoptosis, we decided to reconsider the evidence supporting or challenging this notion. Several reports particularly caught our attention and prompted us to write the current article. For example, in 2001, Geske et al. [8] reported that early stages of p53-induced apoptosis, detected by TUNEL, are reversible. In the same year, Liu et al. [9] demonstrated that apoptosis in tumors of breast cancer patients is associated with poor prognosis. More recently, the TUNEL assay has been used to detect DNA breakage in studies unrelated to cell death [10,11]. These include chromothripsis, which is characterized by extensive genomic rearrangements and provides cells with a growth advantage over those without such rearrangements [10].

Herein, we briefly review: (i) stress-induced responses that are associated with persistent genomic DNA breakage and thus might be detected by the TUNEL assay (Section 2); (ii) reversal of p53-induced apoptosis in the absence of exogenous stress (Section 3); (iii) recovery from the brink of apoptotic death following treatment with anticancer agents (Section 4); and (iv) the generation of growth-stimulating signals by apoptotic cells (Section 5). In light of these discoveries and the TUNEL principle (discussed in Section 6), we reflect on the advantages and drawbacks of the TUNEL assay in preclinical anticancer studies (Section 7) and on the prognostic value of detecting DNA breakage by the TUNEL assay in clinical material (Section 8). Based on the evidence provided, we conclude that caution should be exercised in the interpretation of results obtained by TUNEL and other apoptosis assays in terms of cell death. This cautionary note is particularly relevant to preclinical studies that are performed under conditions that permit cancer cell recovery from the brink of apoptotic death.

## 2. Anticancer Agent-Induced Responses Associated with Persistent Genomic DNA Breakage

As highlighted by Ye et al. [12], “Cellular heterogeneity can alter emergent properties, and cells that diverge from the average population—outliers—often define the direction of cancer evolution. However, cancer researchers have traditionally ignored the contribution of outliers and focused solely on average profiles or dominant clones…under pathological conditions, especially under cellular crisis conditions, some outliers, such as cells with extremely different phenotypes, often become the dominant population.”

**Figure 1:**
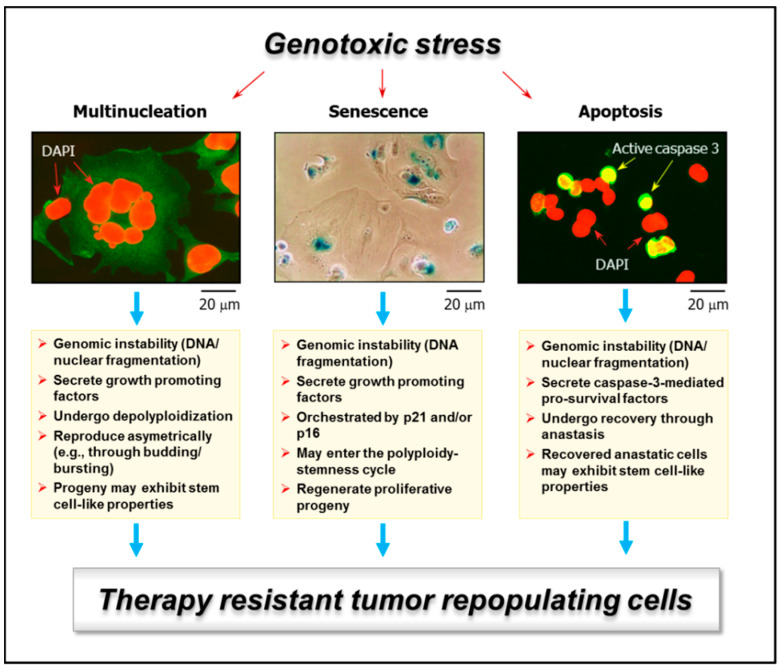
Examples of genotoxic stress-induced responses in solid tumors/tumor-derived cell lines that are often scored as “death” in conventional preclinical assays and yet have the potential of giving rise to tumor-repopulating progeny: multinucleated giant cells (**left**); highly enlarged cells with senescence features (e.g., senescence-associated-β-galactosidase-positive) (**middle**); and apoptotic cells (e.g., active caspase 3-positive) (**right**). Multinucleated giant cells can be created through different routes, including homotypic cell fusions. Stress-induced premature senescence is a genetically-controlled process, mediated by p21^WAF1^ and/or p16^INK4a^, depending on the p53 status of the cells. Apoptosis is also a genetically-controlled process that can lead to cell demise or survival, depending on context. Some features of these responses that can lead to tumor repopulation are indicated. For further details, please consult References [7,18]. DAPI, 4′,6-diamidino-2-phenylindole.

**Figure 2:**
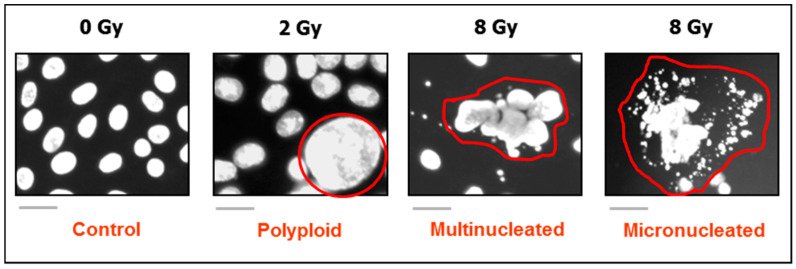
DAPI (4′,6-diamidino-2-phenylindole) images showing the development of giant cells with diverse nuclear abnormalities in cultures of SUM159 breast carcinoma cells after exposure to ionizing radiation and incubation for 3 days. The border of giant cells is marked in red for clarity. Scale bars, 20 µm.

Microscopic observations with solid tumors/tumor-derived cell lines have identified various types of such outlier cells that are often overlooked or scored as “dead” in conventional pre-clinical assays, and yet they remain viable and metabolically active, secrete growth promoting factors, and can give rise to therapy resistant progeny with stem cell-like properties that may repopulate the tumor. Three groups of outliers with these characteristics have been extensively discussed in recent reviews: giant cancer cells with diverse nuclear abnormalities (reviewed in, e.g., References [7,12,13,14,15,16,17,18,19]); cancer cells exhibiting senescence-like features [7,19,20,21,22,23]; and, paradoxically, cancer cells undergoing programmed cell “death” through apoptosis [7,24,25,26,27,28] (also see Figure 1). Giant cells with nuclear abnormalities include those with a highly enlarged nucleus, multiple nuclei, and/or multiple micronuclei (Figure 2). For simplicity, we will refer to such giant cells collectively as polyploid giant cancer cells (PGCCs).

All of these outlier responses are associated with genomic instability, such as DNA strand breakage and nuclear fragmentation. PGCCs, for example, exhibit extensive nuclear fragmentation (e.g., through nuclear budding and bursting [29,30,31]) during their evolution which ultimately leads to the emergence of tumor initiating cells or of tumor repopulating cells post-treatment (reviewed in, e.g., [16,17,18,19]). Cancer cells undergoing stress-induced premature senescence exhibit persistent levels of DNA strand breaks [28,32]; under some circumstances, escape from premature senescence can lead to the formation of PGCCs and PGCC-acquired stemness [14,33,34,35], which involves nuclear fragmentation. In solid tumors, premature senescence and polyploidy/multinucleation can occur simultaneously, e.g., through homotypic cell fusion [36]. Cancer cells triggered to undergo apoptosis also show DNA strand breakage/nuclear fragmentation both at early and late stages of the apoptotic process [8,37,38].

Among these responses, apoptosis has been widely studied for decades in the context of cancer cell radiosensitivity and chemosensitivity, with DNA fragmentation often being used as a marker of apoptosis. Accordingly, the TUNEL assay [39,40] (the principles of which will be outlined below in Section 6) has become the gold standard for detecting cell death through apoptosis. However, as briefly discussed below, TUNEL staining is not always associated with apoptosis, and apoptosis (e.g., detected by TUNEL) is not always associated with cell demise.

## 3. Reversal of p53-Induced Apoptosis in the Absence of Exogenous Stress

Geske and colleagues reported a series of experiments in 2000 [41] and 2001 [8] designed to determine whether early manifestations of apoptosis could be reversed in a mouse mammary carcinoma cell line. The authors used a temperature sensitive p53-containing cell line in which p53 is active at 30 °C and inactive at 37 °C. Incubation of cells at the permissive temperature for 6 h promoted apoptosis, as indicated by phosphatidylserine externalization based on Annexin V staining [8], as well as by DNA fragmentation as visualized by the TUNEL assay [41]. A colony formation assay was used to determine whether cells with these manifestations of apoptosis could survive if the apoptotic stimulus was removed. Cultures that were first incubated at 30 °C for 6 h (to induce manifestations of apoptosis) and then incubated at 37 °C exhibited no loss of colony forming ability when compared to control cultures that were incubated at 37 °C throughout. These observations suggested that early stages of p53-induced apoptosis were reversible in this mouse cell-line model.

As pointed out by these authors [8], “a hypothesis that may explain the reversibility of apoptotic cells is that those selected cells already have undergone genetic alterations which have led to apoptosis resistance.” This possibility was ruled out by demonstrating that cells that had recovered from an initial pulse of apoptotic stimulus (i.e., p53 activation at the permissive temperature) were susceptible to undergoing apoptosis following a second pulse of apoptotic stimulation.

## 4. Recovery from the Brink of Apoptotic Death (Anastasis) Following Treatment with Anticancer Agents

The observation of Geske et al. [8,41] that cells can recover from at least some manifestations of apoptosis went largely unnoticed except for occasional mentions in review articles (e.g., [42]). Since 2009, however, numerous groups have independently reported such a recovery phenomenon in different biological systems, including solid tumor-derived cell lines treated with chemotherapeutic drugs [43,44,45,46,47,48,49,50,51]. The process of recovery from the brink of apoptotic death is now being referred to as anastasis (Greek for “rising to life”) and appears to involve physiological healing processes that could also sustain damaged cells inappropriately, leading to genetic alterations and transformation [26,27,52]. The potential impact of anastasis on the outcome of cancer therapy has been recently discussed [7,26,27,52]. The following observations regarding anastasis should be noted:

- Anastasis has been reported for cancer cell lines expressing wild-type p53 (e.g., MCF7 breast carcinoma; A375 melanoma) [45,51], mutant p53 with gain-of-function properties (e.g., MDA-MB-231 breast carcinoma) [51], and no p53 (e.g., HeLa cervical carcinoma; PC3 prostate carcinoma) [45]. Thus, anastasis does not appear to be dependent on the p53 status of the cells.
- Given that the process of mitochondrial outer membrane permeabilization (MOMP) has been considered to represent the point-of-no-return for many cell types triggered to undergo apoptosis [53], it has been proposed that perhaps anastasis might occur at early stages of apoptosis and only in cells exhibiting low levels of MOMP. Seervi and associates tested this possibility in human cervical (HeLa) and breast (MDA-MB-231) cancer cell lines after treatment with the chemotherapeutic drugs paclitaxel and etoposide [47]. Anastasis was observed not only after “limited” MOMP, i.e., when only a fraction of the mitochondria in a cell were permeabilized and the majority remained intact, but also after “widespread” MOMP, i.e., when most of the mitochondria in a cell were permeabilized.
- In other studies anastasis has been observed in cells that have passed through important checkpoints of apoptosis, including mitochondrial fragmentation, release of mitochondrial cytochrome *c* into the cytosol, activation of caspases, chromatin condensation, DNA breakage, nuclear fragmentation, plasma membrane blebbing, cell shrinkage, cell surface exposure of phosphatidylserine, and formation of apoptotic bodies [45].
- Studies with human breast cancer cell lines (MCF7, T47D, MDA-MB-231) demonstrated that cancer cells that had undergone anastasis after paclitaxel treatment exhibited increased tumorigenicity both in vitro and in vivo when compared with the parental (pretreatment) cells [50,51]. Cancer cells that recover from the brink of death may acquire stem cell-like properties through epigenetic processes, resulting in tumor progression, therapy resistance, and disease recurrence [38,45,49,50,51].
- Recovered cancer cells display an increased number of micronuclei and chromosomal abnormalities that can lead to increased aneuploidy [38,45], a driving force of aggressive disease. It is noteworthy that at least a subset of micronucleated cells can be mistaken for apoptotic cells based on their morphology (nuclear fragmentation; see, e.g., Figure 1 in Reference [15]).

In short, after a few years of inactivity following the original reports of apoptosis reversal by Geske et al. [8,41] in a mouse model, in the past decade, our understanding of this phenomenon in different biological systems and its implications in health and disease has increased at a rapid pace.

## 5. Apoptotic Cells Generate Growth-Stimulating Signals: Implications for Cancer Therapy

In addition to anastasis, other mechanisms of apoptosis-related tumorigenesis are being uncovered [25,54,55,56,57,58,59,60]. These include apoptosis-induced proliferation through caspase-3-mediated production of prostaglandin E_2_, a key regulator of tumor growth. This so-called “Phoenix Rising” pathway was initially shown by Huang et al. [56] to stimulate tumor cell repopulation during cancer radiotherapy. The roles of apoptotic caspases outside apoptosis, such as in stimulating tumor repopulation, epigenetic reprogramming, and carcinogenesis, are now well documented. As stated by Zhao et al. [25], “one important clinical implication of these discoveries is that current anti-oncogenic therapies aimed at activating caspases to kill cancer cells are at best a flawed strategy. In fact, established cancer treatment, such as radiotherapy and chemotherapy, may select for cancer cells that could survive the treatments and become stronger by acquiring new mutations or become more stem cell-like based on sub-lethal caspase activation.”

Secretion of growth-signaling factors by apoptotic cells has been observed without the application of any external stressors (i.e., triggered by self-inflicted DNA breakage [54]), as well as after treatment with external stressors, such as exposure to various doses of ionizing radiation ranging from 0.5 gray (Gy) [58] to 12 Gy [56]. In all cases, apoptotic cells that were shown to secrete growth-promoting factors exhibited persistent DNA breakage. Thus, although not yet reported, it is reasonable to assume that at least a subset of such cells might be positive in the TUNEL assay.

## 6. The Principle of the TUNEL Assay

As noted above, a biochemical hallmark of both the early and late stages of apoptosis is cleavage of the double-stranded genomic DNA by endonucleases, such as caspase-activated DNase (CAD) (also called DNA Fragmentation Factor 40; DFF40), generating small nucleosomal fragments bearing free 3’-OH groups at their termini. Application of the TUNEL methodology to the detection of such DNA fragmentation in apoptotic cells was introduced almost 30 years ago [39,40]. The assay exploits the unique ability of an atypical DNA polymerase, terminal deoxynucleotidyl transferase (TdT), to catalyze the addition of suitably-labeled deoxynucleotide triphosphates (most commonly, dUTP analogs) to a single-stranded sequence with a free 3′-OH terminus in a template-independent manner.

The DNA polymerase that we now know as TdT was initially isolated by Frederick Bollum in 1960 from a calf thymus gland [61]. Several excellent reviews have appeared describing the remarkable properties of this enzyme and should be consulted for details (e.g., [62,63]). Briefly, TdT is a member of the X family of DNA polymerases that typically function in aspects of DNA repair. With a solitary exception, DNA polymerases catalyze the addition of deoxynucleotide 5’-triphosphates to extend a primer sequence using a DNA or RNA template. However, TdT has the remarkable (and to date unique) characteristic of synthesizing short nucleotide chains at the 3′-OH end of a single-stranded DNA primer without the requirement of a template strand. In fact, the presence of a template strand is inhibitory, as discussed further below. Perhaps even more remarkable is its ability to synthesize DNA de novo; thus, TdT has been reported to generate short polynucleotide chains in the absence of any primer sequence [62,63]. Nonetheless, the best-defined activity of TdT, which is normally expressed in immature lymphoid cells, is in adding a few random deoxynucleotides to unpaired 3’-OH-terminal overhangs generated during V(D)J recombination in a template-independent manner. This immunologic activity, which occurs in collaboration with the non-homologous end-joining (NHEJ) DNA repair proteins, serves to increase antigen receptor and antibody diversity.

It should be noted that the TdT protein contains a 16 amino acid “lariat-like” loop that actually inhibits its interaction with a template strand, i.e., with duplex DNA, thus directing its activity towards single-stranded DNA substrates [62,63]. For this reason, the TUNEL assay is generally held to work primarily via the template-independent addition of deoxynucleotides at the 3′-OH termini of double-strand DNA breaks generated during the apoptotic process, with the preferred substrate of TdT being a 3′-OH-terminal single-stranded overhang, although it can also act on blunt or recessed 3′ ends. TdT, however, does appear to be capable of some template-dependent activity [64].

As a result, TdT is capable of labeling any DNA molecule with a suitably accessible 3′-OH terminus independent of how it originated. For this reason, and given the known roles of TdT in DNA repair, notably in NHEJ, the TUNEL assay should not be regarded as specific for apoptotic fragments. In cells undergoing apoptosis following exposure to pharmacologically/clinically-relevant doses of anticancer agents, however, the level of these substrate nucleosomal cleavage fragments with the requisite free 3′-OH termini should be so elevated that these alternative/background signals should have little impact on the ability of the assay to preferentially detect apoptosis-associated events resulting from the treatment per se (although, as noted above, these events may not necessarily lead to cell death). By contrast, in other applications, such as for discriminating low-level signals and/or where no “control” samples are available for comparison (e.g., in prognostic studies using clinical tumor specimens, discussed below in Section 8), it could be more problematic.

## 7. Advantages and Disadvantages of TUNEL in Preclinical Anticancer Studies

Some of the pros and cons of the use of the TUNEL assay in cell-death related studies have been reviewed elsewhere [42]. The advantages include: (i) relative ease of performance when compared to other apoptosis assays; and (ii) availability of TUNEL assay kits for various applications, such as single-cell analysis in cell cultures, flow cytometric analysis of populations of cells, and histochemical and fluorescent analysis of tissue sections and paraffin-embedded samples. Owing to these properties, the TUNEL assay has been the method of choice for assessing apoptosis in various laboratory, animal model, and clinical studies.

The TUNEL assay does have several potential drawbacks for apoptotic cell death assessment [65,66,67,68]. As pointed out by Loo almost a decade ago [67], “because TUNEL staining is nonspecific in the sense that the assay will label all free 3′-hydroxyl termini, irrespective of the molecular mechanisms that led to the development of these termini, TUNEL staining will also detect non-apoptotic cells—including necrotic degenerating cells, cells undergoing DNA repair, cells damaged by mechanical forces, and even cells undergoing active gene transcription. Therefore, TUNEL staining should be considered generally as a method for the detection of DNA damage (DNA fragmentation or others)…” As noted above, the impact of such limitations of the assay will likely depend on the specific application.

Perhaps more importantly, we now know that, as briefly discussed above, several therapy-induced cancer cell responses that are associated with disease relapse might be misinterpreted as “death” by the use of the TUNEL assay. These include: (i) apoptosis in cells that undergo the above-mentioned anastatic recovery process [27]; and (ii) apoptosis in cells that stimulate proliferation of neighboring cells through secreted factors [25].

## 8. The “Gray” Area of TUNEL-Staining Results Obtained in Clinical Prognostic Studies

The availability of commercial TUNEL assay kits that allow the interrogation of large numbers of fresh or preserved clinical tumor samples has seen their broad application to the evaluation of apoptotic “cell death” (apoptotic index) as a prognostic factor in many tumor sites. Reports that have used TUNEL, with or without other apoptosis assays, have yielded variable results. For example, a number of studies have suggested that TUNEL staining of paraffin-embedded tumor tissues is prognostic for poor outcomes in various types of cancer (e.g., [69,70,71]). Other studies show diverse findings even for samples from patients with a single type of cancer, e.g., in non-small cell lung carcinoma (reviewed in Reference [72]). As noted above, one of the problems inherent to these prognostic studies is that they rely on measurements for each patient sample without reference to a “control” sample, such that no allowance can be made for background TUNEL staining/noise. This makes it necessary to develop operational criteria for assigning samples as TUNEL positive (apoptotic) or negative.

The challenges associated with this approach were recognized by Ben-Izhak et al. [73], who assessed apoptosis by immunohistochemical TUNEL staining on paraffin-embedded surgical samples from 73 patients with squamous cell carcinoma of the tongue. No significant relationship between TUNEL staining and 5-year survival was seen. However, the authors concluded that “TUNEL in situ technique for the detection of apoptosis is not completely specific TUNEL evaluation and scoring is a difficult task for clinical application…the margin of error in scoring TUNEL between cases is too narrow to allow for definitive categorization…exact biological pathway expressed by TUNEL staining is yet to be fully elucidated.”

Another complication in such prognostic assessments of apoptosis biomarkers/TUNEL can be seen in an early study by Liu et al. [9]. These investigators examined tumor samples from 791 breast cancer patients using a panel of standard morphological markers of apoptosis; they also evaluated a subset of 234 of the samples for TUNEL staining. High morphological apoptotic counts were associated with poorer survival outcomes and also with various measures of tumor aggressiveness, including tumor grade, tumor size, and lymph node metastasis [9]. Importantly, these authors saw a good agreement between the two apoptosis marker types, implying that TUNEL provides a good representation of what is seen under a microscope. As for why apoptosis might be associated with a more aggressive malignancy, we should first note that the samples interrogated in that study were not stratified by patient treatment history or disease stage, and indeed about a third of the patients were known to have been treated with adjuvant chemotherapy at some unreported point in their management that could have had a profound effect on what was being measured. Nonetheless, there may also be solid biological reasons for this behavior, albeit based on preclinical studies. Thus, as discussed in Section 4, events that would be scored as apoptosis by both the morphological criteria and TUNEL assay used here have the potential to drive tumor progression and aggressiveness if the recently-recognized process of anastasis is in play in in vivo systems, since apoptotic cancer cells that recover and survive can develop increased aneuploidy.

We should stress that the clinical value of a prognostic marker relates more to its robustness and accuracy than its ability to satisfy our mechanistic expectations based on studies in preclinical models. As such, markers such as apoptotic morphology or TUNEL positivity, in a context where they have shown highly significant associations with clinical features, such as tumor stage and grade, are extremely useful in guiding patient management decisions. We would hope, however, that the biological mechanisms that underlie such correlations will eventually become clear and guide future directions in preclinical research.

To this end, there is an urgent need for developing robust biomarkers using in vitro models that will enable the discrimination of cells that exhibit manifestations of apoptosis with respect to whether an individual cell is likely to survive through recovery/anastasis or to undergo terminal apoptotic death [27]. Such biomarkers would enable studies into the occurrence of anastasis in clinical samples and thus into assessing prognosis in a more mechanistically-relevant context, as well as providing critical basic knowledge concerning the incidence of anastasis in different types of human cancer. It is important to note in this context that anastasis is a normal homeostatic mechanism in the in-vivo developmental biology of species, such as Caenorhabditis elegans and Drosophila [27].

## 9. Concluding Remarks

Since 2009, there have been several reviews on cell death that were published in *Cell Death and Differentiation* (e.g., [1,60,74,75]) and other high impact journals (e.g., [25,37,53,76]). Most of these reviews highlighted the dark side of cell “death” in cancer therapy in relation to, e.g., tumor promoting functions of pro-apoptotic factors, such as caspases, p53, and its pro-apoptotic target, PUMA (p53 upregulated modulator of apoptosis). Unfortunately, these articles have largely neglected the impact of the following three key responses in disease recurrence after anticancer treatment: (i) reversal of the apoptotic pathway (anastasis) [26]; (ii) cancer cell dormancy (e.g., through polyploidy, multinucleation, and/or senescence) [7]; and (iii) cancer cell micronucleation associated with genome chaos, a complex cancer cell survival strategy during crisis [13].

With respect to apoptosis, it was demonstrated some twenty years ago that early stage p53-induced apoptosis can be reversed. We now understand that apoptotic cells have the potential for contributing to disease relapse post-therapy by undergoing a reversal process (anastasis); this reversal is observed in cells that exhibit various features of apoptosis, including caspase activation, DNA breakage, and phosphatidylserine externalization. Furthermore, apoptotic cells can promote tumor repopulation through, e.g., the Phoenix Rising pathway that is orchestrated by executioner caspases.

In short, to assume that commonly used apoptosis assays, such as TUNEL, caspase activity, and phosphatidylserine externalization (e.g., annexin V staining), exclusively detect cell death can be misleading, at least in pre-clinical studies. With respect to clinical relevance, whether anastasis (e.g., observed in vitro in TUNEL-positive cells) can occur in cancer cells undergoing apoptosis in their native microenvironment, and if so, whether the recovery will occur in a sufficiently large fraction of apoptotic cancer cells to have biological consequences, including tumor initiation (pre-therapy) and repopulation (post-therapy), remains to be elucidated.

A key take-home message from this short review is that TUNEL remains a powerful tool for assessing manifestations of apoptosis in biological systems, especially when used as part of a panel of complementary biomarkers. However, as new biological concepts, such as the roles of dormancy and anastasis in the response of cancer cells to therapeutic agents, emerge from preclinical studies, it becomes increasingly important to assess their potential impact on how we interpret developments in certain areas, such as cancer prognostic biomarkers and clinical responses to therapeutic agents.

## Abbreviations

NCCD	Nomenclature Committee on Cell Death
TUNEL	Terminal deoxynucleotidyl transferase dUTP nick-end labeling
PGCCs	Polyploid giant cancer cells
MOMP	Mitochondrial outer membrane permeabilization
CAD	Caspase-activated DNase
DFF40	DNA fragmentation factor 40
TdT	Terminal deoxynucleotidyl transferase
NHEJ	Non-homologous end-joining
PUMA	p53 upregulated modulator of apoptosis
DAPI	4′,6-diamidino-2-phenylindole
